# Notoginsenoside R1–Induced Neuronal Repair in Models of Alzheimer Disease Is Associated With an Alteration in Neuronal Hyperexcitability, Which Is Regulated by Nav

**DOI:** 10.3389/fncel.2020.00280

**Published:** 2020-09-04

**Authors:** Tao Hu, Shan Li, Wen-Qi Liang, Shan-Shan Li, Min-Nan Lu, Bo Chen, Li Zhang, Rui Mao, Wan-Hai Ding, Wen-Wei Gao, Shi-Wen Chen, Yan-Bin XiYang, Jie Zhang, Xu-Yang Wang

**Affiliations:** ^1^Department of Laboratory Medicine, The Third People’s Hospital of Yunnan Province, Kunming, China; ^2^Institute of Neuroscience, Basic Medical College, Kunming Medical University, Kunming, China; ^3^Department of Emergency, Shanghai Changhai Hospital, Naval Medical University, Shanghai, China; ^4^Basic Medical College, Experimental Teaching Center, Kunming Medical University, Kunming, China; ^5^Science and Technology Achievement Incubation Center, Kunming Medical University, Kunming, China; ^6^Editorial Department of Journal of Kunming Medical University, Kunming, China; ^7^School of Stomatology, Kunming Medicine University, Kunming, China; ^8^Department of Neurosurgery, Shanghai Jiao Tong University Affiliated 6th People’s Hospital, Shanghai, China; ^9^Yunnan Provincial Key Laboratory for Birth Defects and Genetic Diseases, Department of Medical Genetics, The First People’s Hospital of Yunnan Province, Kunming, China; ^10^Affiliated Hospital of Kunming University of Science and Technology, Kunming, China

**Keywords:** notoginsenoside R1, Alzheimer disease, neuronal hyperexcitability, voltage-gated sodium channel, cognitive impairment

## Abstract

Alzheimer disease is characterized by a progressive cognitive deficit and may be associated with an aberrant hyperexcitability of the neuronal network. Notoginsenoside R1 (R1), a major activity ingredient from *Panax notoginseng*, has demonstrated favorable changes in neuronal plasticity and induced neuroprotective effects in brain injuries, resulting from various disorders, however, the underlying mechanisms are still not well understood. In the present study, we aimed to explore the possible neuroprotective effects induced by R1 in a mouse model of AD and the mechanisms underlying these effects. Treatment with R1 significantly improved learning and memory functions and redressed neuronal hyperexcitability in amyloid precursor protein/presenilin-1 mice by altering the numbers and/or distribution of the members of voltage-gated sodium channels (Nav). Moreover, we determined whether R1 contributed to the regulation of neuronal excitability in Aβ-42–injured cells. Results of our study demonstrated that treatment with R1 rescued Aβ1-42–induced injured neurons by increasing cell viability. R1-induced alleviation in neuronal hyperexcitability might be associated with reduced Navβ2 cleavage, which partially reversed the abnormal distribution of Nav1.1α. These results suggested that R1 played a vital role in the recovery of Aβ1-42–induced neuronal injury and hyperexcitability, which is regulated by Nav proteins. Therefore, R1 may be a promising candidate in the treatment of AD.

## Introduction

Alzheimer disease (AD) is the most typical manifestation of dementia, and its incidence is increasing exponentially. It is prevalent among the elderly and has affected 35 million people worldwide (Ghezzi et al., 2013). AD is characterized by a progressive cognitive decline, which may burden the families of patients as well as healthcare systems. Researchers have been trying to identify drugs that may help slow the progression of AD; however, the results obtained so far have not been satisfactory (Ghezzi et al., 2013). Therefore, developing new drugs for the management of AD is a big unmet medical need.

*Panax notoginseng* is a promising candidate for the management of AD, owing to the multiple types of active components present in this herb. This traditional Chinese herbal medicine is grown in Yunnan and has more than a 100-year history of use in the clinical treatment of a wide range of diseases in China (Ng, 2006; Guo et al., 2010; Kim, 2012). As a major ingredient isolated from *P. notoginseng* saponins (PNSs), notoginsenoside R1 (R1) has been proven to possess anti-inflammatory and antioxidative properties and is useful to reverse damage caused by ischemia–reperfusion (Zhang et al., 1997; Ng, 2006; Zhang and Wang, 2006; Liu et al., 2010; Pang et al., 2017). Recent studies have demonstrated that various ginsenosides contribute to PNS-induced neuroprotective effects in AD (Huang et al., 2014, 2017; Li et al., 2015. By reduction of the amyloid precursor protein (APP) expression, stimulation of α-secretase activity, and inhibition on β-secretase activity, ginsenosides Rb1, Rg1, and Rg3 display protective effects against Aβ production and aggregation (Chen et al., 2006; Wang and Du, 2009; Yang et al., 2009). In addition, a recent study reported that R1 prevented Aβ-induced synaptic dysfunction and improved hippocampal-based memory performance in an AD mouse model through a possible mechanism involved in the modulation of neuronal excitability (Yan et al., 2014). However, the neuroprotection induced by R1 associated with neuronal excitability seen in AD is still not clearly understood.

Voltage-gated sodium channels (Nav) play a crucial role in cell excitability and generate and propagate action potentials (APs) (Hodgkin and Huxley, 1952; Catterall, 2000; Chyung et al., 2005). The main components of Nav include nine α subtypes, namely, Nav1.1 to Nav1.9, and at least one auxiliary β subunit. Nav1.1 is mainly seen in GABAergic interneurons but not in excitatory pyramidal neurons (Yu et al., 2006). The voltage-gated sodium-channel β2 subunit (Navβ2) is indispensable for the generation of normal electric potential and controlling excitability of the neuronal membrane; it also regulates current density and the function of Nav in neurons (Chen et al., 2002; Lopez-Santiago et al., 2006). A previous study conducted by our group demonstrated that the expression of SCN2B (the encoding gene of Navβ2) mRNA was up-regulated in the hippocampus and frontal cortex of senescence-accelerated mice prone 8 (SAMP8). This finding suggested that SCN2B may be closely related to the aging process of the hippocampus and frontal lobe of SAMP8, whereas treatment with Xueshuantong (a trade name of PNS) significantly reversed this trend (XiYang et al., 2016). Administration of PNS also induced an improvement in learning and memory (XiYang et al., 2016). Otherwise, as a novel substrate of BACE1 and γ-secretase, the hydrolyzed Navβ2 expression is significantly increased, and Navβ2 plays an important role in the abnormal intracellular translocation of Nav1.1α and production of Aβ in mice with AD (Hu et al., 2017, 2019). Taken together, these results suggested that the antiaging effect and neuroprotective ability of PNS may be associated with the regulation of Navβ2 expression in the brain in the regions responsible for learning and memory. Our current study presents the first known case of how learning and memory improvement induced by R1, a specific and major ingredient of PNS, is dependent on the alteration of neuronal excitability regulated by Navβ2 in mice with AD.

## Materials and Methods

### Ethics Statement

All experiments and protocols regarding animal care complied with the Guide for the Care and Use of Laboratory Animals published by the US National Institutes of Health (publication no. 85-23; revised 1996). This study was performed in accordance with the Care and Use Guidelines of Experimental Animals established by the Research Ethics Committee of Kunming University of China (permit no. kmu-eac-2018021; Kunming, China). Isoflurane was used to induce anesthesia for surgical procedures. The purchase, storage, and use of isoflurane were approved by Kunming Medical University (license no. kmykdx-D-A00272; Kunming, China).

### Drug Preparation

R1 (C47H80O18, molecular weight 933.16, purity > 99.0%) was purchased from MedChemExpress LLC (CAS no. 80418-24-2, Shanghai, China). R1 was dissolved in dimethyl sulfoxide (DMSO) to yield a 1 mM stock solution and stored at −20°C.

### Animal Grouping

Two-month-old C57BL/6J mice [wild-type (WT) mice] and APPswe/PS1ΔE9 [APP/presenilin-1 (PS1) transgenic mice with a C57BL/6J genetic background] mice were purchased from the laboratory animal center of Kunming Medical University. A previous study demonstrates that APP/PS1 mice show intact cognition at the age of 2 months, learning deficits at 3.5 months, learning and memory deficits at 5 m, and further learning and memory impairments at 8 m (Li et al., 2016). It appears that APP/PS1 mice at 2 months mimic the preclinical stage of AD (prodromal AD), whereas APP/PS1 mice at 8 months exhibit midclinical signs of AD (symptomatic AD) (Li et al., 2016). In order to explore the impact of R1 on mice at the preclinical stage of AD, we used 2-month-old APP/PS1 mice to explore the possible effects of R1 treatment for a consecutive 6-month period to study the age-specific alterations of memory and neuronal excitability related to AD progression. The APP/PS1 mice were randomly divided into the R1-treated group and control group, with each group comprising 15 mice. Animals in the drug-treated group were administered R1 once a day at a dose of 5 mg/kg per day by gavage for 6 months (APP/PS1 + R1–treated group). The control group was treated with an equivalent volume of sterile 10% DMSO instead of R1 (APP/PS1 + vehicle group). Age-matched WT mice were administered an equivalent volume of sterile 10% DMSO, and these mice served as the normal control group (WT + vehicle group, *n* = 15). All experimental mice were housed in wood-chip bedded plastic cages in a 12:12 h light–dark cycle with free access to food and water.

### Novel Object Recognition Task

After 6 months of continuous administration of the study drug, a novel object recognition (NOR) task was performed to evaluate the spatial memory of mice. The task lasted 3 days. A 40 × 40 × 40 cm^3^ transparent plastic box was used to perform this experiment. Previous studies report 24 h as an intertrial interval (ITI) in NOR test when memory function was explored, indicating it to be a promising and reasonable interval for NOR tasks to be performed by APP/PS1 mice (Luo et al., 2020). Therefore, in the present study, we selected 24 h as an ITI for the NOR task. On the first day, a 5-min habituation phase was carried out in the apparatus without introducing any objects. After the 24 h ITI, mice underwent an exploratory phase in the box containing two identical objects for 5 min. On the third day, a novel object (NT) was placed as a substitute for one of the objects in the apparatus. Mice were allowed to move and observe different objects freely; the time spent exploring the NT and familiar object (FT) was recorded. The memory discrimination index (DI), which is considered to be the index of spatial memory ability, was calculated using the following equation: (NT − FT)/(NT + FT).

### Marris Water Maze Test

The Marris Water Maze (MWM) consists of a circular pool (100 cm in diameter, 50 cm deep) filled with white water at 24°C ± 1°C to a depth of 20 cm. The MWM test was run as previously described (XiYang et al., 2016). All mice were subjected to a preliminary experiment, which allowed them to adapt to water and enabled them to step on a white platform (hidden below the water surface) so that they could be rescued. This preliminary experiment was carried out the day before the actual test. Briefly, a mouse placed in water was allowed to swim around for 10 s and then placed on a white platform submerged under water for only 1–2 s. For the regular test, the hidden platform was placed in a different location. The time taken by the mouse to find the hidden submerged white platform was recorded as escape latency and recorded for 5 days in a row. On day 6 of the test, the platform was removed for the probe trial, and each mouse was allowed to swim for 60 s to assess their memory regarding the location of the platform. The amount of time spent in the target quadrant, path taken in the target quadrant, and number of target platform crossings were recorded. The time spent and distances traveled in the four quadrants were also noted. The animals were tracked using an overhead video camera, and the data were analyzed using an animal behavior video-analysis system.

### Electroencephalogram Recordings

For electroencephalogram (EEG) recordings, a high-resolution mouse EEG using polyimide-based microelectrode array (PBM array) was used. Protocols for electrode implantation, EEG recordings, data acquisition, and analysis were performed according to published studies the literature with some modifications (Lee et al., 2011; Corbett et al., 2013; Hu et al., 2017). Mice were anesthetized using isoflurane (2–3% for induction) mixed with O_2_ (0.5–1 L/min) and fixed on the stereotaxic instrument for electrode fixation before subjecting them to EEG recordings. First, a 2 cm incision was made on the middle of the scalp to expose the skull, and the bregma and lambda points were identified. These points are the recognized electrode positions on the surface of the mouse brain on the skull according to the mouse brain atlas (Paxinos and Franklin’s the Mouse Brain in Stereotaxic Coordinates). Then, mice were implanted in the hippocampus (anteroposterior −2.1 mm, mediolateral −1.5, dorsoventral −1.5 mm) with bare three-wire electrodes (1.0 mm in diameter), which consisted of three polyimide-coated wires with bare tips. Two microscrews that were used for ground and reference were fixed on the occipital bone above the cerebellum. Mice implanted with microelectrodes recovered within 5–7 days after surgery. The connector was linked to a multichannel EEG amplifier system, and neural electrical signals were recorded. EEG signals were recorded in freely mobile mice between 10 and 11 a.m., and signal acquisition was carried out in the cages of mice. The original recording signal collected by the recording electrode was amplified 1,000× by the amplifier. Analog signals were digitized at a sampling frequency rate of 1 kHz using a CED Micro1401 (CED, Cambridge, United Kingdom) data acquisition system. The A/D sampling rate was not only 128 Hz, and further, the signal was bandpass-filtered between 1 and 64 Hz. Power spectral densities were generated every 30 s for each recording to calculate the amount of time spent at > 6 Hz (time in high frequency). Signal processing ensured that integer values represented the dominant frequency (DF) in Hz for each 30-s epoch. The mean DF was calculated for each mouse from each DF in each 30-s epoch. Time in high frequency was calculated by summing the number of 30-s epochs with a DF > 6 Hz and dividing it by the total number of 30-s epochs (total time of the recording) for each mouse (Corbett et al., 2013; Hu et al., 2017).

### Western Blot

Mice were sacrificed by cervical dislocation and decapitation following the EEG recording (a week later). Brains were removed and soaked in cold phosphate-buffered saline. Half of the brain was used for Western blot analysis and the rest for cell-surface biotinylation assay.

For Western blot analysis, the brain tissue was separately homogenized using a homogenizer on ice in lysis buffer, which comprised 10% sodium dodecyl sulfate (SDS), 10 mM Tris-HCl buffer (pH 7.4), 30% Triton-1000, 10 mM ethylenediaminetetraacetic acid, protease inhibitor cocktail (Roche, Switzerland), and NaCl. The homogenates were centrifuged at 5,000 *g* for 10 min at 4°C. Proteins were quantified using the bicinchoninic acid reagent (Sigma–Aldrich; Merck KGaA, Darmstadt, Germany) method. Equal amounts of the proteins were separated using SDS–polyacrylamide gel electrophoresis (PAGE) on 4–12% gels. Proteins were then transferred to nitrocellulose membranes and incubated with primary antibodies against BACE1 (1:1,000; cat. no. ab2077; Abcam), Nav1.1α (1:800; cat. no. ASC-001; Alomone), or Navβ2 (1:500; cat. no. ASC-007; Alomone). GAPDH (1:800; cat. no. Sc-365062; Santa Cruz, CA, United States) was used as a loading control. The membranes were subsequently incubated with appropriate secondary antibodies at 20–25°C for 2 h. Horseradish peroxidase–conjugated anti–rabbit antibodies were used for the detection of Nav1.1α and Navβ2 (1:2,000; cat. no. PI-1000; Vector Laboratories, Inc.), whereas GAPDH was detected using a peroxidase-conjugated anti–mouse secondary antibody (1:1,000; cat. no. PI-2000; Vector Laboratories, Inc.). Enhanced chemiluminescence luminol reagent (Beyotime Institute of Biotechnology, Shanghai, China) was used for protein quantification. A densitometry analysis of target protein bands was conducted using a Bio-Rad Gel Imaging System (ChemiDoc^TM^ XRS+; Bio-Rad Laboratories, Inc., Hercules, CA, United States) and Quantity One software v4.6.6 (Bio-Rad Laboratories, Inc.) for each group, in order to quantify the expression levels of proteins.

### Cell-Surface Biotinylation Assay

The remaining brain tissue was placed into ice-cold Krebs solution containing the components described previously (Corbett et al., 2013) with the following modifications: 120 mM NaCl, 4.5 mM KCl, 1.5 mM KH_2_PO_4_, 10 mM glucose, 1.5 mM MgSO_4_, 26 mM NaHCO_3_, and 1.5 mM CaCl_2_. Cell-surface biotinylation and detection of cell-surface Nav1.1α were performed as previously described (Corbett et al., 2013; Hu et al., 2017). NeutrAvidin agarose beads (Pierce, United States) were used to pull down the biotinylated proteins, which were eluted by incubation with SDS–PAGE sample buffer at 37°C for 60 min and analyzed using SDS–PAGE followed by Western blotting as described in section “Western Blot.” For expression analysis of extracellular proteins, biotinylated cell-surface proteins were bound to the beads. The remaining lysate containing non-biotinylated proteins was used for the detection of intracellular proteins of Nav1.1α.

### Cell Culture

Cortical or hippocampal tissues were obtained from postnatal day 0 (P0) C57BL/6J mice as described previously (Kim and Magrane, 2011). The crushed cortical and hippocampal tissues were digested using 1.25% of pancreatin (Gibco) in an incubator at 37°C for 10 min before being resuspended with fetal bovine serum (Gibco). The resulting tissue suspension was filtered using a 70 μm cell strainer to collect the cortical or hippocampal cells. These cells were incubated in six-well culture plates that were precoated with poly-D-lysine (Sigma–Aldrich) and soaked with neuronal medium (Gibco) containing 2% B27 supplement (Gibco), 0.5% penicillin/streptomycin, and 0.25% GlutaMax (Gibco). Half of the culture medium was replaced every 3 days.

### Preparation and Treatment With Aβ1-42

Cultured cells were treated with Aβ1-42 peptide to mimic Aβ-induced neuronal injury. The Aβ1-42 peptide (Sigma–Aldrich) was dissolved in 1,1,1,3,3,3- hexafluoro-2-propanol to yield a concentration of 1 mM. After the Aβ1-42 peptide was incubated in the fume hood at room temperature for 24 h, a clear peptide film was formed, which was stored at −20°C. The Aβ1-42 peptide was resuspended in DMSO. The cultured cortical and hippocampal cells were treated using 5 μM Aβ1-42 peptide (preparation described in section “Cell Culture”), which was diluted in neural basal medium for 5 h.

### Cell Treatment

We observed that R1 administration induced neuronal restoration in APP/PS1 mice, improved learning and memory, and was useful in redressing in brain hyperexcitatory. In order to identify whether R1 contributed to the regulation of neuronal excitability Aβ1-42–injured cultured cells were treated using 5 μM R1 (named as R1 group). Aβ1-42–injured cells that were treated with DMSO served as one of the control groups (DMSO group), whereas those treated with medium served as the blank (blank group). The cultured cells untreated by Aβ1-42 and PNS monomers were also used as baseline control. Upon establishment of these treatment protocols, the cultured cells were used for further evaluation.

### 3-(4,5-Dimethylthiazol-2-yl)-2,5-Diphenyltetrazolium Bromide Assay

Cell viability was measured using a 3-(4,5-dimethylthiazol-2-yl)-2,5-diphenyltetrazolium bromide (MTT) assay kit per manufacturer’s guidelines (Sigma–Aldrich). Briefly, the primary neuronal cells were seeded in 96-well plates at a density of 2 × 10^4^ cells per well and treated with various exogenous substances according to the followed protocol. Following incubation, MTT reagent was added to the cells at 37°C. After incubation for 4 h, the culture medium was replaced with 100 μL DMSO to dissolve the formazan crystals. The absorbance of each well was measured at 570 nm by using a microplate reader (Microplate Reader, Bio-Rad, 3550).

### Electrophysiological Patch Clamp Recording

Previous studies have suggested that increased neuronal hyperexcitability induced by Aβ1-42 is associated with an increased sodium current (Ciccone et al., 2019). Hence, we investigated whether the regulation of sodium current by R1 affected neuronal excitability. Electrophysiological measurements were used to detect APs or sodium currents of the primary cortex or hippocampal neurons at room temperature. Electrophysiological patch clamp recording was performed using a previously described method (Shan et al., 2019). Briefly, cells were patched in voltage clamp mode and kept at −70 mV. The membrane capacitance (Cm), input resistance (Rm), and the time constant (tau) were calculated by applying the depolarization step voltage command (mV) and using the pClamp10 software integration test function of the membrane. Next, recordings were switched to current clamp mode. The resting film potential was adjusted to −80 mV by introducing a positive current (50–100 pA). A series of depolarizing current pulses was then used, and intrinsic excitation was checked by constructing the input–output (i–o) function.

### Statistical Analysis

Statistical analyses were performed using SPSS 19.0 (IBM, Armonk, NY, United States) for Windows covariance software package. The data are expressed at mean ± standard deviation (SD). Student’s *t-*test was used to determine significance between two groups. Statistical differences among multiple groups were evaluated using analysis of variance (ANOVA) and Bonferroni *post hoc* test. Two-way repeated-measures (RM) ANOVA followed by Tukey test was used for the analysis of results from the MWM test. NOR data were analyzed using two-way ANOVA. EEG data were analyzed using one-way ANOVA followed by Student’s *t*-test for paired groups (two tailed). *p* < 0.05 was considered to be statistically significant.

## Results

### R1 Improved Cognitive Impairment of APP/PS1 Mice

**FIGURE 1:**
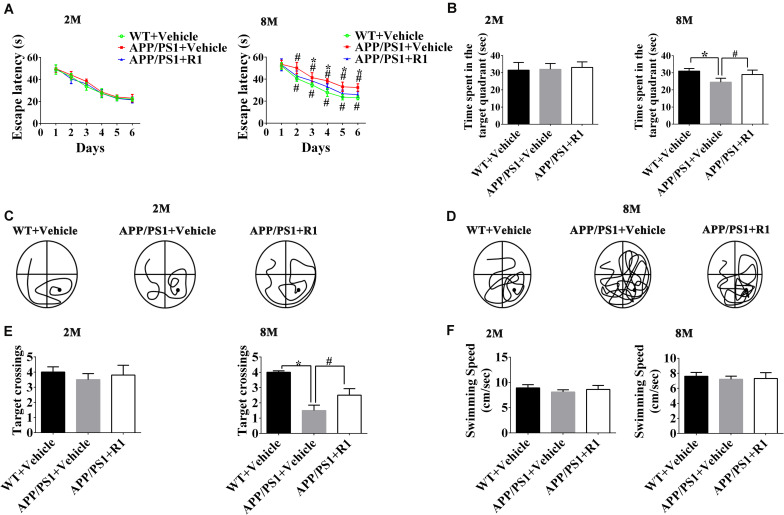
Effects of R1 on spatial learning and memory changes in APP/PS1 mice as indicated by the Morris water maze test. **(A)** Escape latency time of mice in the training period from days 1 to 6. **(B)** Time spent in the target quadrant during the probe test. Swimming paths of 2-month-old **(C)** and 8-month-old **(D)** APP/PS1 mice during the probe test. **(E)** Entries and crossings through the target platform position during the probe test. **(F)** Average swimming speed of mice during probe trial. Data are expressed as mean ± SD, *n* = 15 mice/group. *p < 0.05 vs. WT+ vehicle, ^#^*p* < 0.05 vs. APP/PS1 + R1.

The effects of R1 on the learning and memory functions of APP/PS1 mice were evaluated by using the NOR task and MWM test. Two-way RM ANOVA revealed that the escape latency progressively decreased over time in all groups (WT + vehicle, APP/PS1 + vehicle, and APP/PS1 + R1 group). There was no significant difference in the escape latency between 2-month-old APP/PS1 mice and 2-month-old WT mice. However, the 8-month-old APP/PS1 mice spent more time to find the hidden platform compared to the age-matched WT mice, while R1 significantly shrank the difference (*p* < 0.05, Figure 1A). The platform was removed during the probe test. The 2-month-old mice did not display statistically significant differences in the number of target crossings, the time spent in the target quadrant, and percentages of path in the target quadrant (*p* > 0.05, Figures 1B,C,E). The 8-month-old APP/PS1 mice spent shorter time in the target quadrant, showed lesser number of target crossings, and had lower percentages of path in the target quadrant compared to those of the aged-matched WT mice (*p* < 0.05, Figures 1B,D,E). As shown in Figure 1F, the average swimming speed between the WT and APP/PS1 mice showed no significant difference (*p* > 0.05, Figure 1F). Treatment with R1 partially improved the MWM test behavior in APP/PS1 mice (Figures 1B–E).

**FIGURE 2:**
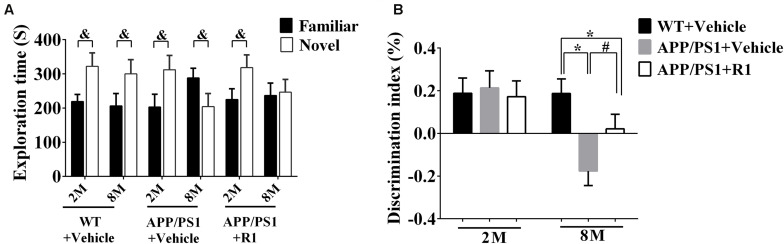
Effects of R1 on NOR tasks in APP/PS1 mice. **(A)** Comparison of the exploration time on familiar objects and novel objects in APP/PS1 mice. **(B)** Discrimination Index obtained from different groups. Data are expressed as mean ± SEM, *n* = 15 mice/group. *p < 0.05 VS. WT + vehicle, ^#^*p* < 0.05 vs. APP/PS1 + R1, and ^&^*p* < 0.05 vs. novel.

The discrimination index obtained from the NOR task demonstrated that there was no significant difference between the WT and 2-month-old APP/PS1 mice in the task exploring new objects (*p* > 0.05, Figure 2B). The 8-month-old APP/PS1 mice spent less time in the NOR task compared to the age-matched WT mice (*p* < 0.05) (Figure 2B). Treatment with R1 increased the time taken by mice in the NOR task in the APP/PS1 group (APP/PS1 + R1 vs. APP/PS1 + vehicle, *p* < 0.05) (Figures 2A,B).

Taken together, the above results indicated that R1 could be a beneficial compound for improving spatial learning and memory loss in transgenic mice with AD (Figures 1, 2).

### R1 Reversed Abnormal Neuronal Hyperexcitability of APP/PS1 Mice and Regulated the Expression of BACE1 and Nav

**FIGURE 3:**
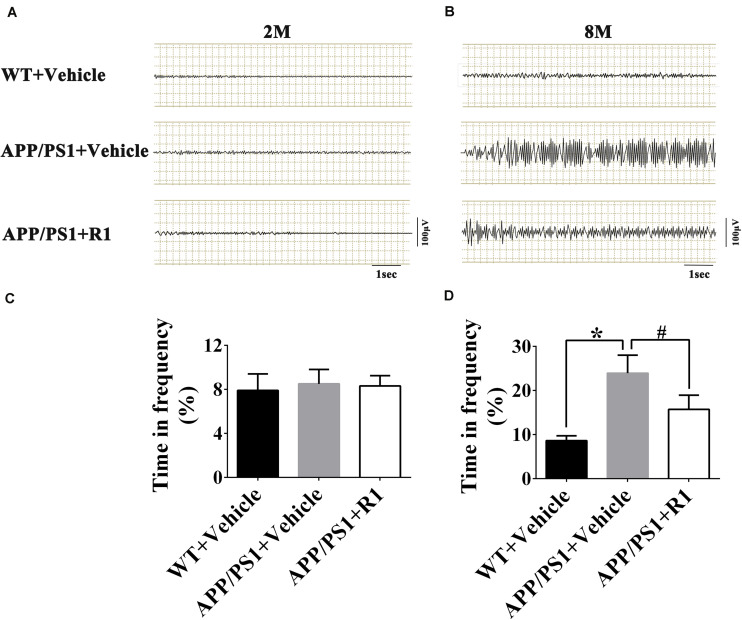
Notoginsenoside R1 administration induced improvement in neuronal hyperexcitability and abnormal neural activity detected using EEG. Spike-wave discharges of APP/PS1 mice, APP/PS1 mice + R1, and APP/PS1 mice + vehicle in 2 m **(A)** and 8 m **(B)**, and the time in high frequency (%) obtained from different groups in 2-month-old **(C)** and 8-month-old mice **(D)** (*n* = 15), *p < 0.05 vs. WT+ Vehicle, ^#^*p* < 0.05 vs. APP/PS1 + R1.

To investigate the possible effects of R1 on the aberrant neuronal hyperexcitability, EEG was recorded in WT and APP/PS1 mice following various treatments. The EEGs of the 8-month-old APP/PS1 mice showed increased spike-wave discharges and time in high frequency significantly compared to those in the age-matched WT mice (Figure 3). Although the 8-month-old APP/PS1 mice treated with R1 exhibited ameliorative neuronal excitability, it did not reach the level of the age-matched WT mice (Figure 3). These results indicated that R1 treatment improved neuronal hyperexcitability induced by APP/PS1 mutation.

**FIGURE 4:**
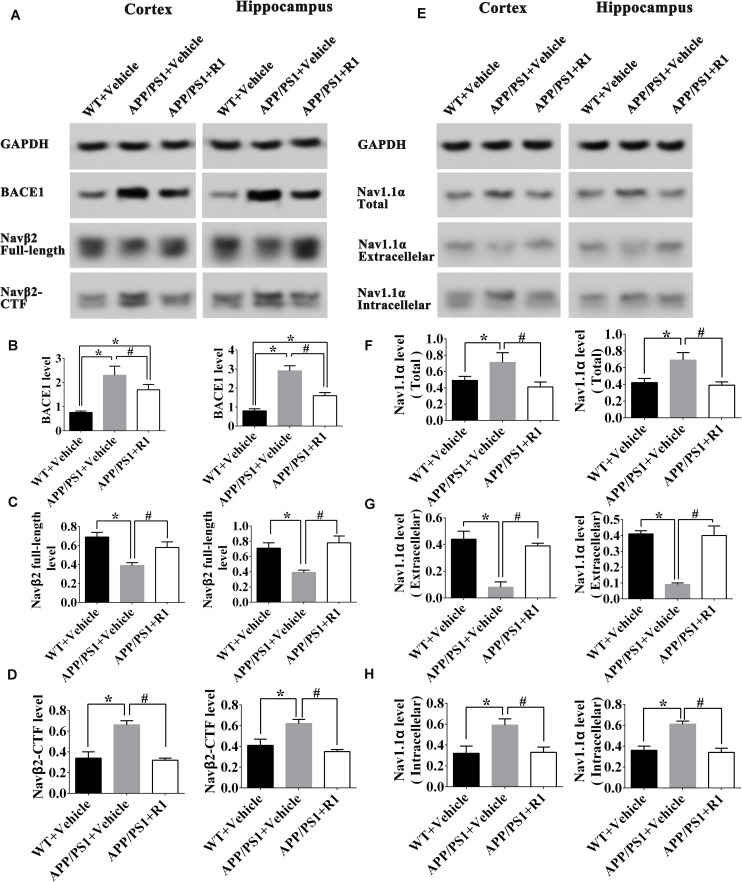
Notoginsenoside R1 altered the distribution of Nav1.1α and cleavage of Navβ2 in the brains of APP/PS1 mice. Representative expression **(A,E)** and densitometric quantification of BACE1 **(B)**, Navβ2 full-length **(C)**, Navβ2-CTF **(D)**, total expression of Nav1.1α **(F)** extracellular Nav1.1α **(G)**, and intracellular Nav1.1α **(H)** protein in the cortex and hippocampus lysates from WT mice, APP/PS1 mice, or APP/PS1 + R1 mice, respectively. Data are shown as mean ± SD (*n* = 15). *p < 0.05 vs. WT + vehicle, ^#^*p* < 0.05 vs. APP/PS1 + R1.

Next, we explored the mechanisms underlying ameliorative neuronal excitability. The crucial roles of the Nav family members and BACE1 in neuronal excitability regulation, the levels of BACE1, enzymolysis status of Navβ2, and the expression and locations of Nav1.1α were determined. R1 treatment significantly decreased the expression of BACE1 in the cortex and hippocampus of APP/PS1 mice (APP/PS1 + R1 vs. APP/PS1 + vehicle, *p* < 0.05) (Figures 4A,B). Simultaneously, increased full-length Navβ2 and decreased Navβ2-CTF fragments were detected in APP/PS1 mice treated with R1 (APP/PS1 + R1 vs. APP/PS1 + vehicle, *p* < 0.05) (Figures 4A,C,D). These results also demonstrated that the abnormal extracellular and intracellular distributions of Nav1.1α in the cortex or hippocampus were redressed using R1 (APP/PS1 + R1 vs. APP/PS1 + vehicle, *p* < 0.05, Figures 4E–H). Taken together, these data suggested that R1-induced alleviation in neuronal excitability might be associated with a reduction in Navβ2 cleavage, which partially reversed the abnormal distribution of Nav1.1α in the cortex and hippocampus.

### The Effects of R1 on Cell Viability of Aβ1-42–Induced Injured Neurons

**FIGURE 5:**
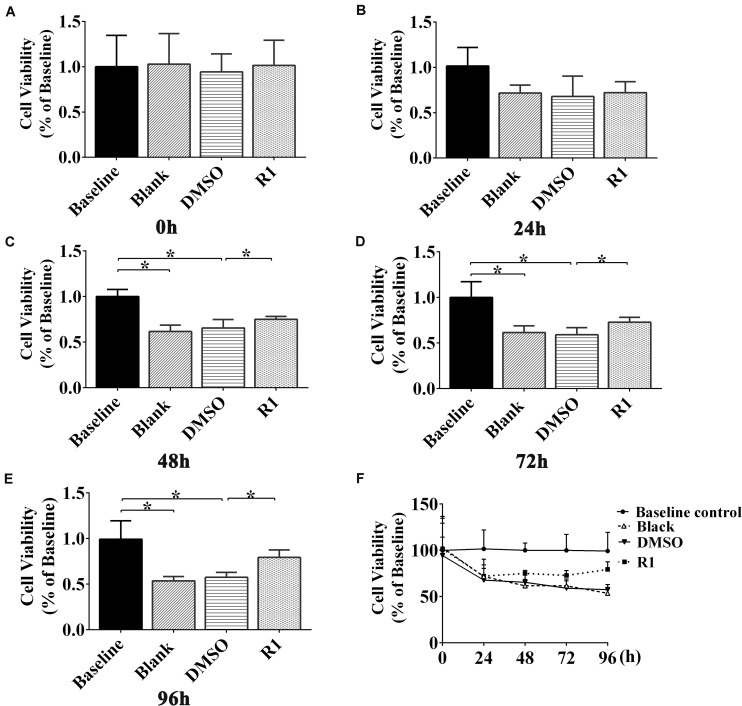
Effect of R1 on Aβ1-42–induced neuron viability determined using MTT assay. Cell viability of cultured neurons treated with different groups at 0 h **(A)**, 24 h **(B)**, 48 h **(C)**, 72 h **(D)**, and 96 h **(E)**. **(F)** Cell viability of cultured neurons in innocent neurons (baseline control), neurons treated with Aβ1-42 (blank group), and Aβ1-42–treated neurons incubated with DMSO (DMSO group) or R1 (R1 group), at different time points. Data are shown as mean ± SD (*n* = 5). *p < 0.05.

The cell viability of Aβ1-42 (5μM)–injured cultured cortical and hippocampal neurons was measured using an MTT assay at 0, 24, 48, 72, and 96 h posttreatment with R1. Our results demonstrated that there was no significant difference between the DMSO-treated and blank control groups (Figures 5A–E). The cellular activity of the blank group significantly decreased when compared to that of baseline control group at 48,72, and 96 h posttreatment (*p* < 0.05) (Figures 5C–E). These results demonstrated that treatment with Aβ1-42 led to a significant damage in neurons after 48 h of incubation. Compared to the DMSO group, treatment with R1 induced significant recovery in cell viability in Aβ1-42–injured cells (*p* < 0.05) (Figures 5C–E). We observed that R1-treated neurons recovered from injury and showed the highest cellular activity at 96 h (Figure 5F). Therefore, the above results confirmed that R1 had therapeutic effects on Aβ1-42–induced neuronal damage.

### Effects of R1 on the Neuronal Hyperexcitability and Sodium Current Overload Induced by Aβ1-42

**FIGURE 6:**
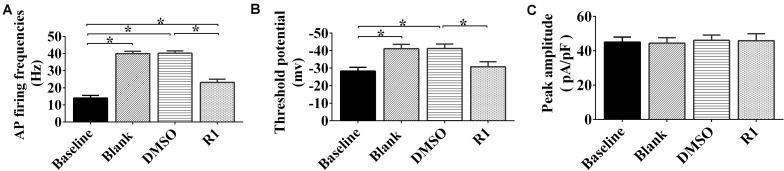
Effects of notoginsenoside R1 on neuronal hyperexcitability induced by Aβ1-42. Changes in the action potential frequency **(A)**, threshold **(B)**, and peak amplitude **(C)** in neurons after R1 treatment. Data are shown as mean ± SD (*n* = 9). *p < 0.05.

To determine whether R1 contributed to the redressal of neuronal hyperexcitability induced by Aβ1-42, whole-cell patch-clamp recordings were performed on cultured neurons after application, following different treatment protocols. We found that the frequency of neuronal APs after treatment with Aβ1-42 significantly increased compared to the baseline control group (*p* < 0.05) (Figure 6). There was also a significant decrease in the threshold (*p* < 0.05) (Figures 6A,B). The frequency, threshold, and peak amplitude of the neuronal AP in the DMSO-treated group showed no significant difference when compared to the blank control (*p* > 0.05) (Figures 6A–C). These results demonstrated that model of neuronal hyperexcitability induced by Aβ1-42 was reliable and also consistent with a previously published study (Wang et al., 2016).

In Aβ1-42–injured neurons, treatment with R1 induced a decrease in frequency of neuronal AP and increase in threshold potential when compared to that of DMSO-treated neurons (*p* < 0.05, Figures 6A,B). It was notable that the threshold potential detected in the R1-treated groups showed no statistically significant difference when compared to that of the baseline group (*p* > 0.05) (Figure 6B). Moreover, the peak current density showed no significant difference after R1 treatments (*p* > 0.05) (Figure 6C). These results suggested that R1 was responsible for reducing abnormal excitability in Aβ1-42–induced neurons.

**FIGURE 7:**
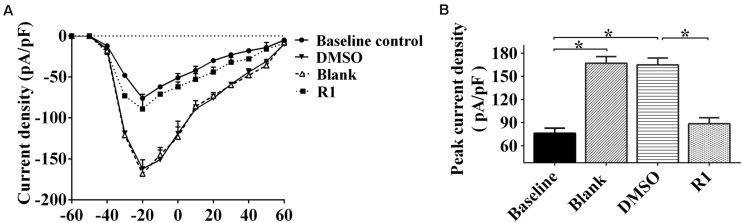
Effects of R1 on the changes of neuronal sodium current. **(A)** Current–voltage relationships of sodium current density in innocent neurons (baseline control) and neurons treated with Aβ1-42 (blank group), Aβ1-42–treated neurons cultured with DMSO (DMSO group), or R1 (R1 group). **(B)** Peak current densities in Aβ1-42–injured neurons after various treatments. Data are shown as mean ± SD (*n* = 9). *p < 0.05.

Electrophysiological patch clamp recording was used to determine whether neuronal excitability was related to increased sodium current. We found that sodium current density curves and current density of neurons significantly increased after treatment with Aβ1-42 when compared to the untreated baseline control group (Figure 7A). The parameters of sodium current in the DMSO group were not significantly different compared to those of the blank group (*p* > 0.05) (Figures 7A,B). The current and peak current densities of R1-treated groups were decreased when compared to those of the DMSO-treated group (*p* < 0.05, Figures 7A,B). These results indicated that R1 contributed to restore the Aβ1-42–induced neuronal sodium current overload.

### Effects of R1 on the Aberrant Status of Sodium-Channel Proteins Induced by Aβ1-42

To understand the underlying mechanism by which R1 redressed neuronal hyperexcitability and sodium-channel overload caused by Aβ1-42, we examined the expression, location, and hydrolysis status of sodium-channel proteins including Nav1.1α, Navβ2, and BACE1 in Aβ1-42–injured neurons.

**FIGURE 8:**
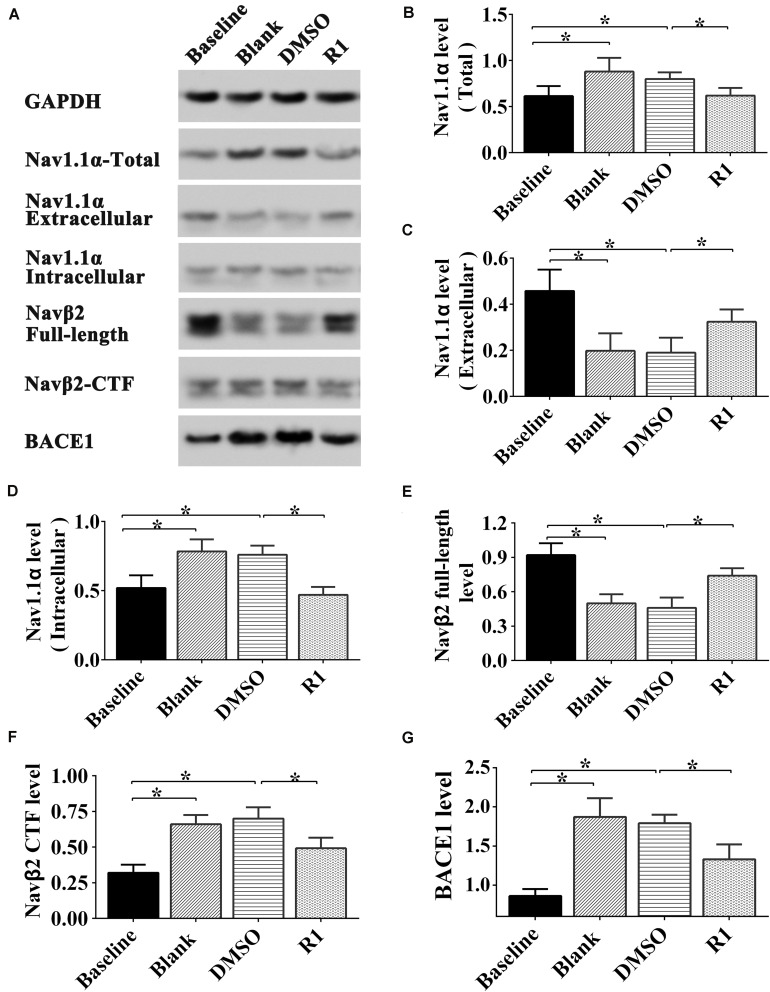
Effect of R1 on the expression, location, and cleavage status of sodium channel proteins after Aβ1-42 treatment. Representative Western blot **(A)** and densitometry quantification of the total Nav1.1α **(B)**, extracellular Nav1.1α **(C)**, intracellular Nav1.1α **(D)**, Navβ2 full-length **(E)**, Navβ2-CTF **(F)**, and BACE1 **(G)** expression. Data are shown as mean ± SD (*n* = 5). *p < 0.05.

As shown in Figure 8, after Aβ1-42 treatment, the expression of total Nav1.1α, intracellular Nav1.1α, Navβ2-CTF, and BACE1 significantly increased compared to that in the baseline control group (*p* < 0.05) (Figures 8A,B,D,F,J). The full-length Navβ2 decreased in the blank and DMSO-treated groups compared to that in the baseline control group (*p* < 0.05) (Figures 8A,E). Moreover, the abnormal release of intracellular Nav1.1α and expression was observed in Aβ1-42–treated groups, which explained the abnormal activation of BACE1 induced by Aβ1-42 (blank group vs. baseline control group, *p* < 0.05) (Figures 8A,C,D,J). Additionally, the expression of Nav protein was not significantly different between the blank control and DMSO-treated groups (*p* > 0.05) (Figures 8A–K).

Among the PNS monomer–treated groups, the levels of total, intracellular, and extracellular Nav1.1α in the R1-treated group were statistically different in comparison to those of the DMSO group (*p* < 0.05) (Figures 8B–D), however, it showed no statistical difference compared to that of the baseline control group in total and intracellular Nav1.1α levels (*p* > 0.05) (Figures 8B,D). Meanwhile, we observed increased Navβ2 full-length levels and reduced Navβ2-CTF in the R1-treated group when compared to the DMSO-treated group (*p* < 0.05) (Figures 8E,F). The levels of full-length Navβ2 and Navβ2-CTF in the R1-treated group showed a statistically significant difference in comparison to those of the baseline control group (*p* < 0.05) (Figures 8J,K). Additionally, a significant decrease in the level of BACE1 was detected in the R1-treated group (*p* < 0.05, Figure 8J). Taken together, these results suggested that the R1-induced alleviation of neuronal excitability might be associated with the reduction in Navβ2 cleavage and partial reversal of the abnormal distribution of Nav1.1α.

The above results collectively demonstrated that neuroprotection induced by R1 was associated with the recovery in the abnormal distribution of Nav1.1α and the abnormal activation of BACE1 induced by Aβ1-42.

## Discussion

Previous studies have demonstrated that by binding to the effects of anti-inflammatory properties, reducing blood viscosity, and antiapoptosis, ginsenosides treatment has outstanding advantage in therapy of neurological disorders accompanied with cognitive impairment (Liu et al., 2014, 2018; Sheng et al., 2014; Zhong et al., 2015). For example, individuals with a commonly occurring brain disorder, traumatic brain injury, characterized by intracranial/intracerebral hemorrhage (Regan et al., 2018), generally develop symptoms of cognitive deficit (Ratliff et al., 2020). Researchers report that administration of PNS reduced inflammation, increased hematoma absorption in patients with acute ICH, and improved behavioral function (Gao et al., 2012). Furthermore, treatment with ginsenosides remarkably improved cognitive performance in patients with AD and in rodent models (Lee et al., 2008; Liu et al., 2012). In this study, we identified that R1 had the ability to regulate sodium-channel expression and thus redressed abnormal neuronal excitability, which otherwise contributes to brain damage and cognitive impairment in AD.

Our study results also identified that R1 was involved in regulating Nav proteins and redressing aberrant neuronal hyperexcitability induced by Aβ1-42. R1 treatment significantly improved learning and spatial memory function and ameliorated abnormal brain excitability in APP/PS1 mice. Consistent with these findings, R1 administration also induced the inhibition of BACE1 activity, thus reducing the excessive cleavage of Navβ2 and correcting the abnormal expressions and distribution of Nav1.1α. It also reduced the neuronal hyperexcitability induced by APP/PS1 mutations. These results suggested that neuroprotection exerted by R1 in APP/PS1 model might be associated with the recovery of neuron hyperexcitability regulated by Nav proteins. Our results revealed that R1 was effective in improving neuronal viability and excitability in Aβ1-42–injured neurons. R1 treatment also restored the voltage-gated sodium current and reduced neuronal excitability by regulating the distribution of Nav1.1α and expression of Navβ2.

We observed that the impairment of spatial learning and memory functions and the paradoxical discharge of brain were significantly improved in APP/PS1 mice following R1 treatment. These results suggested that the corrected intrinsic neuronal hyperexcitability might contribute to cognitive recovery after treatment with R1. Previous studies have reported that XST administration significantly improved spatial memory in APP/PS1 mice, which suggests improvements in neuroplasticity and function (Huang et al., 2018). A previous study indicates that APP/PS1 mutation-induced brain injury and cognitive impairment may be caused by changes in neuronal activity (Leonard and McNamara, 2007). Our findings confirmed that administration of R1 in APP/PS1 mice corrected neuronal hyperexcitability. In order to determine signaling mechanisms, Aβ1-42–injured primary cultured neurons of mice were used as an *in vitro* AD model to depict neuronal hyperexcitability injury. Previous studies have demonstrated that soluble Aβ1-42 oligomers play a key role in neuronal hyperactivity, subsequent cognitive deficits, and memory dysfunction in AD (Bakker et al., 2012; Busche et al., 2012). These findings were consistent with results obtained in our study.

As demonstrated in our study, treatment with R1 rescued cell viability after incubation with Aβ1-42. R1 treatment reduced the frequency of AP and increased its threshold in Aβ1-42–injured neurons. The above results suggested the neuroprotective effect of R1 in Aβ1-42–injured neurons. Previous studies have demonstrated multiple mechanisms of R1 in the treatment of AD (Wang et al., 2013). For example, treatment with R1 provided neuroprotection to APP/PS1 mice by increasing the levels of insulin-degrading enzymes (Li et al., 2015). Moreover, R1 has previously been reported to improve cognitive function in AD mice and may be involved in the regulation of neuroexcitability; these reports are consistent with our conclusions (Yan et al., 2014). The results of our study revealed a molecular mechanism of R1-induced improvement in the learning and memory functions in the mouse model of AD.

To investigate the mechanisms underlying the improved cognitive functions and neuronal excitability, we measured the activity of BACE1 in the cortex and hippocampus of mice. Previous studies demonstrated that increased levels of BACE1, Navβ2 cleavage fragments (Navβ2-CTF), and associated aberrations in Nav1.1α expression and localization were observed in the cortical lysates of APP transgenic mice (Corbett et al., 2013). Therefore, we proposed that the modulation of neuronal excitability by R1 in the AD model was associated with the regulation of the expression of Nav channels. Previous studies showed that the knockdown of Navβ2 partially reversed the excessive degradation of Navβ2 and aberrant intracellular translocation of Nav1.1α. The knockdown also restored electrical excitability of hippocampal neurons and significantly improved learning and memory functions in transgenic mice (Hu et al., 2017, 2019). Studies have also demonstrated that increased Nav1.1α levels are associated with cognitive deficits in mice (Corbett et al., 2013). BACE1 inhibition is known to effectively reduce the production of Aβ, which proves to be a valuable therapeutic strategy in AD (Cheret et al., 2013; Filser et al., 2015; Zhu et al., 2018). We found that R1 treatment significantly improved cell viability, reduced the cleavage of Navβ2 by BACE1 suppression, and also corrected the abnormal distribution of Nav1.1α. These results also showed that administration of R1 regulated sodium current by regulating the enzymatic hydrolysis of Navβ2. Therefore, R1 administration might prove to be a promising therapeutic strategy for the treatment of AD.

## Conclusion

Our results demonstrated that administration of R1 significantly promoted neuronal repair and improved cognition in mice with AD by modulating neuronal excitability. R1 improved abnormal neuronal hyperexcitability and memory deficit and was associated with the regulation of Nav proteins. Next, we will test the learning and memory function change between APP/PS1 mice with or without R1 prolonged administration to further understand whether the neuroprotective effect could last or not after the 6-month R1 treatment in APP/PS1 mice.

## Data Availability Statement

The raw data supporting the conclusions of this article will be made available by the authors, without undue reservation, to any qualified researcher.

## Ethics Statement

The animal study was reviewed and approved by the Committee of Kunming University of China (permit no. kmu-eac-2018021; Kunming, China).

## Author Contributions

TH, SL, W-QL, S-SL, X-YW, Y-BX, and JZ designed the study, performed analyses, and prepared the manuscript. TH, SL, W-QL, W-HD, S-SL, M-NL, BC, W-WG, S-WC, X-YW, Y-BX, JZ, and BC conducted animal experiments. TH, SL, W-QL, S-SL, M-NL, BC, W-HD, LZ, RM, X-YW, Y-BX, JZ, and BC performed cell culture, cell transfection, and cell treatment. TH, W-QL, W-WG, Y-BX, SL, X-YW, and JZ conducted the electrophysiology evaluation. TH, S-SL, Y-BX, W-QL, S-WC, SL, LZ, and RM finished the statistical analyses. All authors were substantially involved in the research, acquisition of data, analysis and manuscript preparation and have read and approved the final submitted manuscript.

## Conflict of Interest

The authors declare that the research was conducted in the absence of any commercial or financial relationships that could be construed as a potential conflict of interest.

## References

[B1] Bakker A. Krauss G. L. Albert M. S. Speck C. L. Jones L. R. Stark C. E. (2012). Reduction of hippocampal hyperactivity improves cognition in amnestic mild cognitive impairment. *Neuron* 74 467–474. 10.1016/j.neuron.2012.03.023 22578498 PMC3351697

[B2] Busche M. A. Chen X. Henning H. A. Reichwald J. Staufenbiel M. Sakmann B. (2012). Critical role of soluble amyloid-beta for early hippocampal hyperactivity in a mouse model of Alzheimer’s disease. *Proc. Natl. Acad. Sci. U.S.A.* 109 8740–8745. 10.1073/pnas.1206171109 22592800 PMC3365221

[B3] Catterall W. A. (2000). From ionic currents to molecular mechanisms: the structure and function of voltage-gated sodium channels. *Neuron* 26 13–25. 10.1016/s0896-6273(00)81133-81132 10798388

[B4] Chen C. Bharucha V. Chen Y. Westenbroek R. E. Brown A. Malhotra J. D. (2002). Reduced sodium channel density, altered voltage dependence of inactivation, and increased susceptibility to seizures in mice lacking sodium channel beta 2-subunits. *Proc. Natl. Acad. Sci. U.S.A.* 99 17072–17077. 10.1073/pnas.212638099 12481039 PMC139271

[B5] Chen F. Eckman E. A. Eckman C. B. (2006). Reductions in levels of the Alzheimer’s amyloid beta peptide after oral administration of ginsenosides. *FASEB J.* 20 1269–1271. 10.1096/fj.05-5530fje 16636099

[B6] Cheret C. Willem M. Fricker F. R. Wende H. Wulf-Goldenberg A. Tahirovic S. (2013). Bace1 and Neuregulin-1 cooperate to control formation and maintenance of muscle spindles. *EMBO J.* 32 2015–2028. 10.1038/emboj.2013.146 23792428 PMC3715864

[B7] Chyung J. H. Raper D. M. Selkoe D. J. (2005). Gamma-secretase exists on the plasma membrane as an intact complex that accepts substrates and effects intramembrane cleavage. *J. Biol. Chem.* 280 4383–4392. 10.1074/jbc.M409272200 15569674

[B8] Ciccone R. Franco C. Piccialli I. Boscia F. Casamassa A. de Rosa V. (2019). Amyloid beta-Induced Upregulation of Nav1.6 Underlies Neuronal Hyperactivity in Tg2576 Alzheimer’s Disease Mouse Model. *Sci. Rep.* 9:13592 10.1038/s41598-019-50018-50011 PMC6753212 31537873

[B9] Corbett B. F. Leiser S. C. Ling H. P. Nagy R. Breysse N. Zhang X. (2013). Sodium channel cleavage is associated with aberrant neuronal activity and cognitive deficits in a mouse model of Alzheimer’s disease. *J. Neurosci.* 33 7020–7026. 10.1523/jneurosci.2325-12.2013 23595759 PMC6618875

[B10] Filser S. Ovsepian S. V. Masana M. Blazquez-Llorca L. Brandt Elvang A. Volbracht C. (2015). Pharmacological inhibition of BACE1 impairs synaptic plasticity and cognitive functions. *Biol. Psychiatry* 77 729–739. 10.1016/j.biopsych.2014.10.013 25599931

[B11] Gao L. Zhao H. Liu Q. Song J. Xu C. Liu P. (2012). Improvement of hematoma absorption and neurological function in patients with acute intracerebral hemorrhage treated with Xueshuantong. *J. Neurol. Sci.* 323 236–240. 10.1016/j.jns.2012.09.028 23062408

[B12] Ghezzi L. Scarpini E. Galimberti D. (2013). Disease-modifying drugs in Alzheimer’s disease. *Drug Des. Devel. Ther.* 7 1471–1478. 10.2147/dddt.S41431 24353405 PMC3862506

[B13] Guo H. B. Cui X. M. An N. Cai G. P. (2010). Sanchi ginseng (*Panax notoginseng* (Burkill) F. H. Chen) in China: distribution, cultivation and variations. 57 453–460. 10.1007/s10722-010-9531-9532

[B14] Hodgkin A. L. Huxley A. F. (1952). A quantitative description of membrane current and its application to conduction and excitation in nerve. *J. Physiol.* 117 500–544. 10.1113/jphysiol.1952.sp004764 12991237 PMC1392413

[B15] Hu T. Li S. S. Lu M. N. Zhang L. Chen B. Mao R. (2019). Neuroprotection induced by Navbeta2knockdown in APP/PS1 transgenic neurons is associated with NEP regulation. *Mol. Med. Rep.* 20 2002–2011. 10.3892/mmr.2019.10406 31257483

[B16] Hu T. Xiao Z. Mao R. Chen B. Lu M. N. Tong J. (2017). Navbeta2 knockdown improves cognition in APP/PS1 mice by partially inhibiting seizures and APP amyloid processing. *Oncotarget* 8 99284–99295. 10.18632/oncotarget.21849 29245901 PMC5725092

[B17] Huang J. Wu D. Wang J. Li F. Lu L. Gao Y. (2014). Effects of Panax notoginseng saponin on alpha, beta, and gamma secretase involved in Abeta deposition in SAMP8 mice. *Neuroreport* 25 89–93. 10.1097/wnr.0000000000000048 24165110

[B18] Huang J. L. Jing X. Tian X. Qin M. C. Xu Z. H. Wu D. P. (2017). Neuroprotective Properties of Panax notoginseng Saponins via Preventing Oxidative Stress Injury in SAMP8 Mice. *Evid. Based Complement. Alternat. Med.* 2017:8713561. 10.1155/2017/8713561 28250796 PMC5303860

[B19] Huang Y. Guo B. Shi B. Gao Q. Zhou Q. (2018). Chinese herbal medicine xueshuantong enhances cerebral blood flow and improves neural functions in Alzheimer’s disease mice. *J. Alzheimers Dis.* 63 1089–1107. 10.3233/jad-170763 29710701 PMC6004915

[B20] Kim H. J. Magrane J. (2011). Isolation and culture of neurons and astrocytes from the mouse brain cortex. *Methods Mol. Biol.* 793 63–75. 10.1007/978-1-61779-328-8_4 21913093

[B21] Kim J. H. (2012). Cardiovascular diseases and panax ginseng: a review on molecular mechanisms and medical applications. *J. Ginseng. Res.* 36 16–26. 10.5142/jgr.2012.36.1.16 23717100 PMC3659571

[B22] Lee M. Kim D. Shin H. S. Sung H. G. Choi J. H. (2011). High-density EEG recordings of the freely moving mice using polyimide-based microelectrode. *J. Vis. Exp.* 47:e2562. 10.3791/2562 21248705 PMC3182650

[B23] Lee S. T. Chu K. Sim J. Y. Heo J. H. Kim M. (2008). Panax ginseng enhances cognitive performance in Alzheimer disease. *Alzheimer Dis. Assoc. Disord.* 22 222–226. 10.1097/WAD.0b013e31816c92e6 18580589

[B24] Leonard A. S. McNamara J. O. (2007). Does epileptiform activity contribute to cognitive impairment in Alzheimer’s disease? *Neuron* 55 677–678. 10.1016/j.neuron.2007.08.014 17785172

[B25] Li X. Y. Men W. W. Zhu H. Lei J. F. Zuo F. X. Wang Z. J. (2016). Age- and Brain Region-Specific Changes of Glucose Metabolic Disorder, Learning, and Memory Dysfunction in Early Alzheimer’s Disease Assessed in APP/PS1 Transgenic Mice Using (18)F-FDG-PET. *Int. J. Mol. Sci.* 17:1707. 10.3390/ijms17101707 27763550 PMC5085739

[B26] Li Z. Li H. Zhao C. Lv C. Zhong C. Xin W. (2015). Protective Effect of Notoginsenoside R1 on an APP/PS1 Mouse Model of Alzheimer’s Disease by Up-Regulating Insulin Degrading Enzyme and Inhibiting Abeta Accumulation. *CNS Neurol. Disord. Drug Targets* 14 360–369. 10.2174/1871527314666150225141521 25714973

[B27] Liu H. Liang J. P. Li P. B. Peng W. Peng Y. Y. Zhang G. M. (2014). Core bioactive components promoting blood circulation in the traditional Chinese medicine compound xueshuantong capsule (CXC) based on the relevance analysis between chemical HPLC fingerprint and in vivo biological effects. *PLoS One* 9:e112675. 10.1371/journal.pone.0112675 25396725 PMC4232446

[B28] Liu J. Yan X. Li L. Zhu Y. Qin K. Zhou L. (2012). Ginsennoside rd attenuates cognitive dysfunction in a rat model of Alzheimer’s disease. *Neurochem. Res.* 37 2738–2747. 10.1007/s11064-012-0866-862 22903450

[B29] Liu W. J. Tang H. T. Jia Y. T. Ma B. Fu J. F. Wang Y. (2010). Notoginsenoside R1 attenuates renal ischemia-reperfusion injury in rats. *Shock* 34 314–320. 10.1097/SHK.0b013e3181ceede4 20023602

[B30] Liu Y. Liu T. Ding K. Liu Z. Li Y. He T. (2018). Phospholipase Cgamma2 signaling cascade contribute to the antiplatelet effect of Notoginsenoside Fc. *Front. Pharmacol.* 9:1293. 10.3389/fphar.2018.01293 30459626 PMC6232503

[B31] Lopez-Santiago L. F. Pertin M. Morisod X. Chen C. Hong S. Wiley J. (2006). Sodium channel beta2 subunits regulate tetrodotoxin-sensitive sodium channels in small dorsal root ganglion neurons and modulate the response to pain. *J. Neurosci.* 26 7984–7994. 10.1523/jneurosci.2211-06.2006 16870743 PMC6674206

[B32] Luo Y. Yang W. Li N. Yang X. Zhu B. Wang C. (2020). Anodal Transcranial Direct Current Stimulation Can Improve Spatial Learning and Memory and Attenuate Aβ(42) Burden at the Early Stage of Alzheimer’s Disease in APP/PS1 Transgenic Mice. *Front. Aging Neurosci.* 12:134. 10.3389/fnagi.2020.00134 32595486 PMC7239315

[B33] Ng T. B. (2006). Pharmacological activity of sanchi ginseng (*Panax notoginseng*). *J. Pharm. Pharmacol.* 58 1007–1019. 10.1211/jpp.58.8.0001 16872547

[B34] Pang H. H. Li M. Y. Wang Y. Tang M. K. Ma C. H. Huang J. M. (2017). Effect of compatible herbs on the pharmacokinetics of effective components of Panax notoginseng in Fufang Xueshuantong Capsule. *J. Zhejiang Univ. Sci. B* 18 343–352. 10.1631/jzus.B1600235 28378572 PMC5394099

[B35] Ratliff W. A. Mervis R. F. Citron B. A. Schwartz B. Rubovitch V. Schreiber S. (2020). Effect of mild blast-induced TBI on dendritic architecture of the cortex and hippocampus in the mouse. *Sci. Rep.* 10:2206 10.1038/s41598-020-59252-59254 PMC7010659 32042033

[B36] Regan S. L. P. Knight P. G. Yovich J. L. Arfuso F. Dharmarajan A. (2018). Growth hormone during in vitro fertilization in older women modulates the density of receptors in granulosa cells, with improved pregnancy outcomes. *Fertil. Steril.* 110 1298–1310. 10.1016/j.fertnstert.2018.08.018 30503129

[B37] Shan L. Galaj E. Ma Y. Y. (2019). Nucleus accumbens shell small conductance potassium channels underlie adolescent ethanol exposure-induced anxiety. *Neuropsychopharmacology* 44 1886–1895. 10.1038/s41386-019-0415-417 31096263 PMC6784903

[B38] Sheng S. Wang Y. Long C. Su W. Rong X. (2014). Chinese medicinal formula Fufang Xueshuantong capsule could inhibit the activity of angiotensin converting enzyme. *Biotechnol. Biotechnol. Equip.* 28 322–326. 10.1080/13102818.2014.911611 26019516 PMC4433917

[B39] Wang X. Zhang X. G. Zhou T. T. Li N. Jang C. Y. Xiao Z. C. (2016). Elevated neuronal excitability due to modulation of the voltage-gated sodium channel Nav1.6 by Abeta1-42. *Front. Neurosci.* 10:94. 10.3389/fnins.2016.00094 27013956 PMC4783403

[B40] Wang Y. Feng Y. Fu Q. Li L. (2013). Panax notoginsenoside Rb1 ameliorates Alzheimer’s disease by upregulating brain-derived neurotrophic factor and downregulating Tau protein expression. *Exp. Ther. Med.* 6 826–830. 10.3892/etm.2013.1215 24137274 PMC3786787

[B41] Wang Y. H. Du G. H. (2009). Ginsenoside Rg1 inhibits beta-secretase activity in vitro and protects against Abeta-induced cytotoxicity in PC12 cells. *J. Asian Nat. Prod. Res.* 11 604–612. 10.1080/10286020902843152 20183297

[B42] XiYang Y. B. Wang Y. C. Zhao Y. Ru J. Lu B. T. Zhang Y. N. (2016). Sodium Channel Voltage-Gated Beta 2 Plays a Vital Role in Brain Aging Associated with Synaptic Plasticity and Expression of COX5A and FGF-2. *Mol. Neurobiol.* 53 955–967. 10.1007/s12035-014-9048-9043 25575679

[B43] Yan S. Li Z. Li H. Arancio O. Zhang W. (2014). Notoginsenoside R1 increases neuronal excitability and ameliorates synaptic and memory dysfunction following amyloid elevation. *Sci. Rep.* 4:6352. 10.1038/srep06352 25213453 PMC4161968

[B44] Yang L. Hao J. Zhang J. Xia W. Dong X. Hu X. (2009). Ginsenoside Rg3 promotes beta-amyloid peptide degradation by enhancing gene expression of neprilysin. *J. Pharm. Pharmacol.* 61 375–380. 10.1211/jpp/61.03.0013 19222911

[B45] Yu F. H. Mantegazza M. Westenbroek R. E. Robbins C. A. Kalume F. Burton K. A. (2006). Reduced sodium current in GABAergic interneurons in a mouse model of severe myoclonic epilepsy in infancy. *Nat. Neurosci.* 9 1142–1149. 10.1038/nn1754 16921370

[B46] Zhang H. S. Wang S. Q. (2006). Notoginsenoside R1 from Panax notoginseng inhibits TNF-alpha-induced PAI-1 production in human aortic smooth muscle cells. *Vascul. Pharmacol.* 44 224–230. 10.1016/j.vph.2005.12.002 16458614

[B47] Zhang W. J. Wojta J. Binder B. R. (1997). Notoginsenoside R1 counteracts endotoxin-induced activation of endothelial cells in vitro and endotoxin-induced lethality in mice in vivo. *Arterioscler. Thromb. Vasc. Biol.* 17 465–474. 10.1161/01.atv.17.3.465 9102164

[B48] Zhong L. Zhou X. L. Liu Y. S. Wang Y. M. Ma F. Guo B. L. (2015). Estrogen receptor alpha mediates the effects of notoginsenoside R1 on endotoxin-induced inflammatory and apoptotic responses in H9c2 cardiomyocytes. *Mol. Med. Rep.* 12 119–126. 10.3892/mmr.2015.3394 25738436 PMC4438911

[B49] Zhu K. Xiang X. Filser S. Marinkovic P. Dorostkar M. M. Crux S. (2018). Beta-Site Amyloid Precursor Protein Cleaving Enzyme 1 Inhibition Impairs Synaptic Plasticity via Seizure Protein 6. *Biol. Psychiatry* 83 428–437. 10.1016/j.biopsych.2016.12.023 28129943

